# Morphological and Morphometric Analysis of the Orbital Aperture and Their Correlation With Age and Gender: A Retrospective Digital Radiographic Study

**DOI:** 10.7759/cureus.17739

**Published:** 2021-09-05

**Authors:** Apurba Patra, Rajan K Singla, Manoj Mathur, Priti Chaudhary, Anjali Singal, Adil Asghar, Vishal Malhotra

**Affiliations:** 1 Anatomy, All India Institute of Medical Sciences, Bathinda, IND; 2 Anatomy, Government Medical College, Patiala, IND; 3 Radiology, Government Medical College, Patiala, IND; 4 Anatomy, Orthopaedics, All India Institute of Medical Sciences, Patna, IND; 5 Family and Community Medicine, Government Medical College, Patiala, IND

**Keywords:** morphology, digital radiograph, dimensions, ethnicity, orbit

## Abstract

Purpose

Precise knowledge about clinically observed bony orbital aging is needed for surgical planning for acceptable cosmetic results. The effect of age and gender on the facial skeleton and orbital aperture has been appreciated earlier, but its quantification remains ignored. The purpose of this study was to evaluate age- and sex-related changes in the shape of the orbital aperture and construct a reference data set for the aging phenomenon in Indians.

Methods

Two hundred digital radiographs (Water’s/frontal view) of the skull, obtained for various reasons, were evaluated. The radiographs comprised 107 males and 93 females aged between 10 and 60 years (10-59 years). Orbital shape, height/width, and interorbital/biorbital distances were noted, and orbital indices (OIs) were calculated. Orbital parameters thus obtained were compared between right and left sides and males and females. The relation of the parameters with age and gender was analyzed.

Results

Four types of orbits, round (33.5%), elliptical (30.5%), rectangular (27.5%), and square (9.5%), were noted in the study population. The average value of height and width of the right orbit was found to be higher than that of the left (p > 0.05). Male patients had higher (p > 0.05) and wider (p > 0.05) orbits than females. The right OI (81.55 ± 5.30) was higher than the left (80.75 ± 4.80) (p > 0.05). When comparatively evaluated between gender, both orbits were found to be of the microseme type with a mere difference (p > 0.05). The average interorbital/biorbital distance was 1.27 ± 2.11 and 9.78 ± 4.40 cm, respectively, without any gender difference. No significant relation was found between the age change and the parameters defined (p > 0.05), except in one age group (10-19 years).

Conclusions

Orbital dimensions showed no association with age and gender except in one age group (10-19 years); a pubertal growth spurt in females might be causing this phenomenon. The morphometric data may be useful in forensic anthropology and better planning for reconstructive surgeries in the orbito-maxillary region.

## Introduction

The orbital cavities are situated on both sides of the sagittal section of the skull, in between the cranium and facial skeleton. Orbital anatomy is vital for clinical assessment and treatment of ocular pathologies. Moreover, physical anthropologists use these traits to explore population relationships, trace population origins, and examine human variation and evolution. Studies have shown that the role of the bony orbit in determining population affinity cannot be substituted by any other cranial or facial trait [1].

Accurate knowledge about the bony orbit is desirable at multiple stages of cosmetic surgery. Identification and quantification of deviations from a well-defined standard allow judgment of the expected bony and soft-tissue contributions for resection and reconstruction. The lack of images of the prepathological stage of the affected orbit forced clinicians to understand normal deviation for optimization of the outcome. Quantitative evaluation of the bony orbit is an integral part of craniofacial surgery and syndromology [2]. Standardized baseline data depending upon age, biological sex, and race or ethnicity are worthwhile because these reflect the potentially different patterns of craniofacial growth resulting from differences in age, sex, and race or ethnicity [3].

The facial features undergo dynamic changes not only in soft tissue but also in facial bones. Many reports are available that have examined the changes in bones of the midface. The bones of the midface, including bones of the orbit, undergo resorption and volume loss with age [1]. Pessa et al. [4] reported the curvilinear enlargement of the orbit with age. Kim et al. [5] demonstrated an age-associated increase in width of the orbital aperture. Researchers who have studied the orbit in various populations found different values of its dimensions in different age groups. Patnaik et al. [6] stated that the orbital width is usually greater than the height and the relation between the two, as given by the orbital index (OI = orbital height/orbital width × 100) varies with age, sex, and ethnic group. Taking the OI as the standard, three classes of the orbit have been described.

1. Megaseme (large): the OI is 89 or over. This type is seen in yellow races [7].

2. Mesoseme (intermediate): the OI ranges between 89 and 83. This type is seen in the white races.

3. Microseme (small): the OI is 83 or less. This type is characteristic of the black races where the orbital opening is rectangular [7].

Knowledge of this index is crucial in various aspects, such as inspection of the fossil record, skull categorization in forensic medicine, scrutinizing the trends in evolution, and ethnic differences. Furthermore, documented ranges of OI may help in distinguishing skulls among different ethnic populations [8]. Most researchers have studied the dimensions of the orbital rim on dry skulls and in human cadavers [9]. Additionally, few researchers have used plain radiographs of the skulls for data accuracy [10,11].

In the present study, we have examined plain digital radiographs of the skull (frontal view), primarily to evaluate the various types of shapes of the orbits, their height, and width, along with interorbital/biorbital distance. The secondary aim was to define the effects of age and gender on orbital morphology and morphometry.

## Materials and methods

The study was carried out on plain digital radiographs of the frontal view of the skull. Owing to the image magnification properties of the X-ray machine, the images obtained are slightly non-identical from those obtained from direct measurements of dry human skulls. Therefore, to minimize these differences and to obtain a valid reproducible radiograph, the angle of emission of radiation, the distance from the source, and the positioning of the patient have to be standardized [5]. This can be attained by placing the chin of the patient on the X-ray cassette with the canthomeatal line (the line that connects the lateral canthus and the external auditory meatus) at 37° to 45°. In the case of the frontal view of the skull, this orientation was accomplished by placing the nose of the patient approximately 0.5 to 1.5 cm above the X-ray plate.

**Figure 1:**
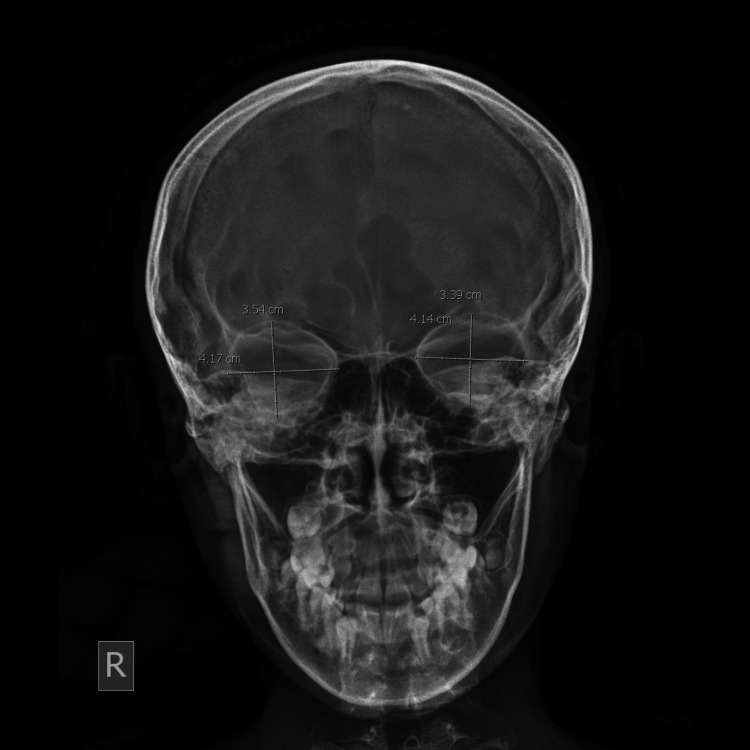
Frontal digital radiograph of skull showing the orbital margin and measurement of orbital height and width.

Two hundred radiographs of 107 males and 93 females, aged between 10 and 59 years were analyzed over two years. Therefore, a total of 400 orbital margins were measured (200 from each side). Frequencies of distribution of age and sexes are shown in Table 1. Measurements were taken only on normal radiographs. Cases with a history of diseases causing raised intracranial or intraorbital pressure, bone diseases, and fractures skulls were excluded from the study. The measurements were taken on a digital monitor. All images were evaluated and interpreted by the same radiologist. Standard anatomical points were determined and used for the measurement of the orbital height and width (Figure 1).

**Table 1:** Frequency of age and sex distributions among the study population.

Age group (in years)	Males (n = 103)	Females (n = 97)	Combined (n = 200)
10-19	3	2	5
20-29	35	28	63
30-39	38	33	71
40-49	23	22	45
50-59	9	7	12
Total	107	93	200

**Figure 2:**
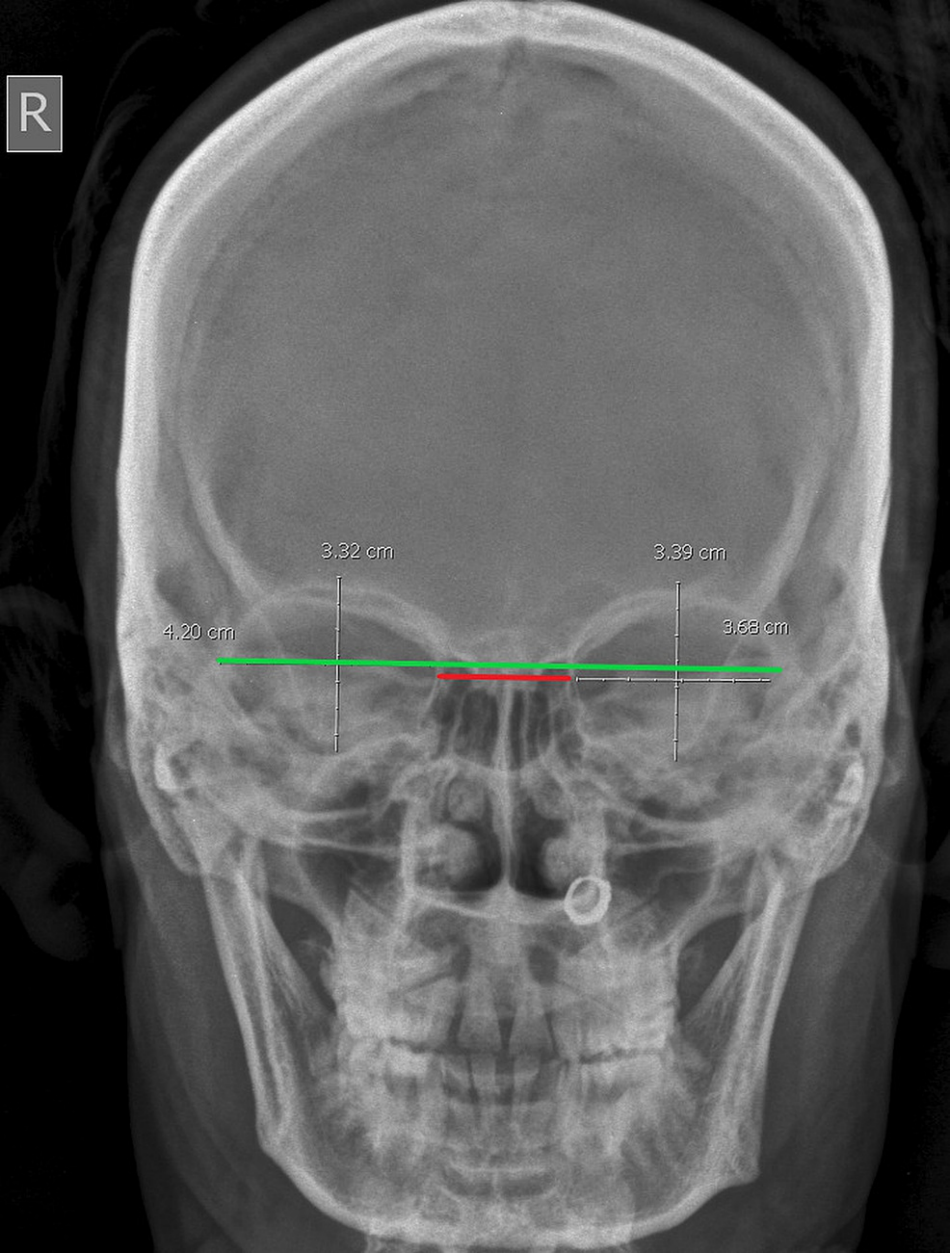
Frontal radiograph showing measurements of interorbital/biorbital distances; red line: interorbital distance; green line: biorbital distance.

The orbital height was measured from the frontal film as the distance between the superior and inferior orbital margins; it is perpendicular to its width and similarly divides the orbit into two halves. The orbital width was also measured from the same frontal film as the distance between the midpoint of the medial margin of the orbit to the midpoint of the lateral margin. The OIs were calculated from the data collected using the following formula: OI = (height of orbit/width of the orbit) × 100. The interorbital distance, that is, the minimum distance between the medial walls of the orbits (distance between the left and right dacryon), and biorbital distance, that is, the maximum distance between the lateral walls of the orbits (distance between right and left ectoconchion), were also measured (Figure 2).

The results were displayed as mean ± standard deviation and analyzed using the SPSS 16.0 software online trial version for Windows (SPSS Inc., Chicago, IL, USA). A p-value less than 0.05 (p < 0.05) was considered significant.

## Results

**Figure 3:**
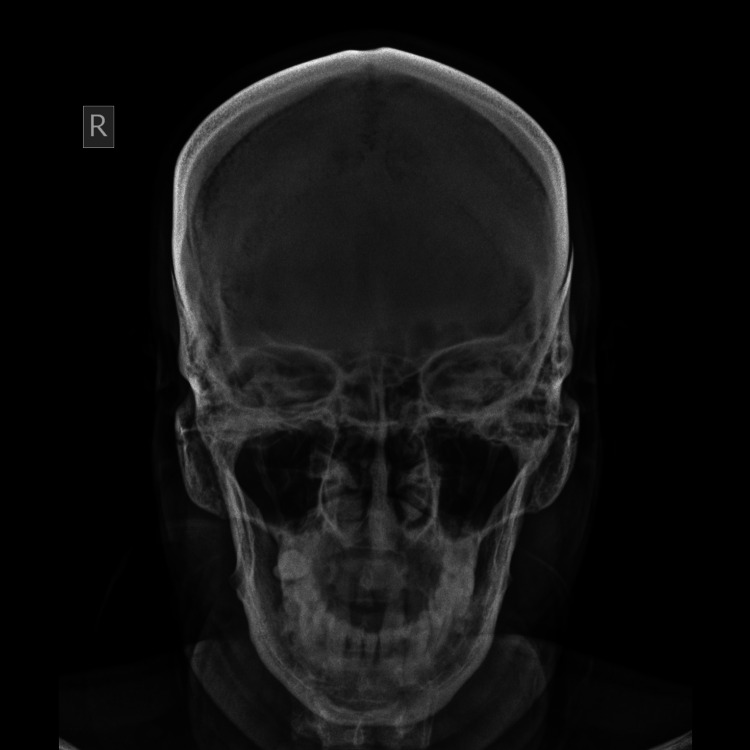
Transversely elliptical orbit.

**Figure 4:**
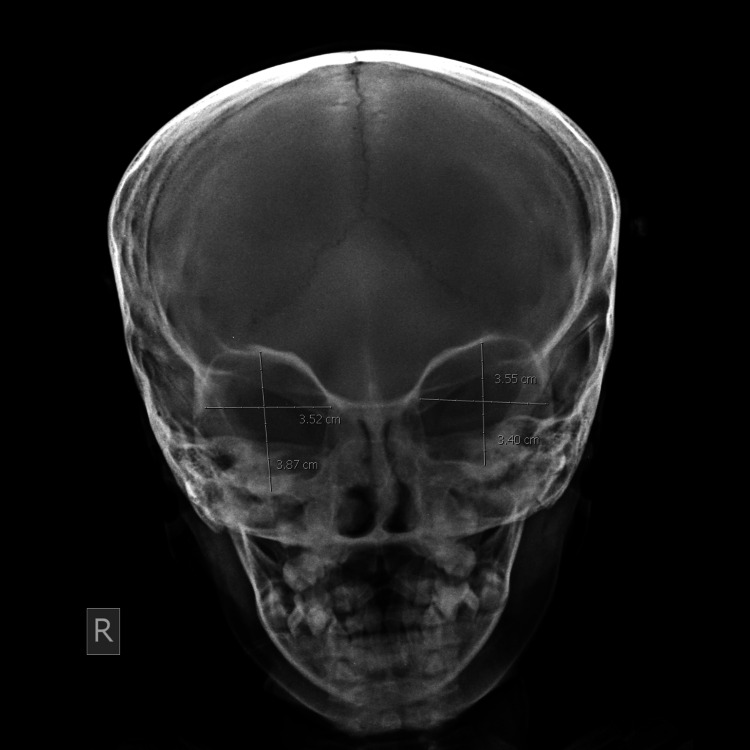
Rectangular orbit.

**Figure 5:**
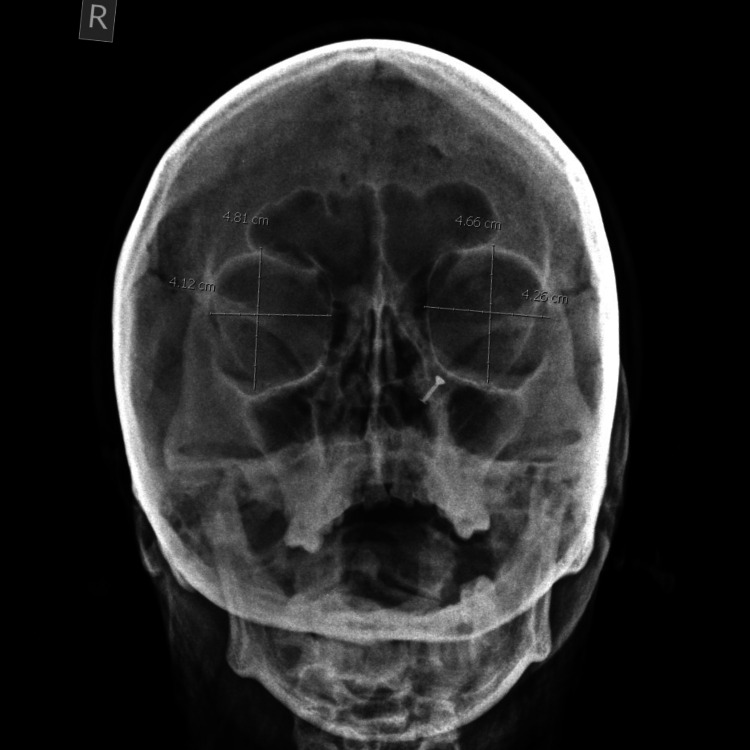
Square-shaped orbit.

Two hundred frontal radiographs were analyzed morphologically and morphometrically. Of the patients, 107 (53.5%) were male and 93 (46.5%) were female. The mean age was 38 ± 11.20 years in males and 42 ± 12.30 years in females, and the combined mean age was 41 ± 12.30 years. The age difference between the genders was non-significant. Gross examination showed mainly four types of orbits in the study population: round orbits were found most commonly (33.5%), closely followed by elliptical (30.5%) (Figure 3), rectangular (27.5%) (Figure 4), and square (9.5%) (Figure 5).

Round orbits were mostly found in lower age groups (10-29 years). As the age advances, gradual changes in shape were noted from round to elliptical in the age group of 30-49 years and becoming rectangular in the later age group (50-59 years). Variations were also observed in the contours of the orbit. The inferior contour of the orbit, formed by the maxilla and zygoma, showed maximum variability, followed by the medial margin. The superior contour, formed by the frontal bone, showed the least variability. Orbital shape showed a significant gender difference in all age groups except in the 10-19 years age group.

The height and width of the orbits were as follows: mean right orbital height was 3.40 ± 1.80 cm, and mean width was 4.31 ± 2.04 cm; mean left orbital height was 3.23 ± 1.40 cm, and mean width was 4.27 ± 2.02 cm. When right and left orbital heights and widths were statistically analyzed, the right orbit was higher and wider than the left, but the difference was insignificant (p > 0.05). Similarly, when right and left orbital heights and widths were comparatively evaluated between gender groups, right and left orbital heights were larger in males than in females, but the difference was again insignificant (p > 0.05). It was also found that right and left orbital widths were larger in males than in females but statistically not significant (p > 0.05) (Table 2). The OI was 81.5 5 ± 5.30 for the right-side orbit and 80.75 ± 4.80 for the left side orbit. When right and left OIs were compared, the right-side OI was found to be higher than that of the left side, but the difference was statistically insignificant (p > 0.05). When right and left OIs were comparatively evaluated between gender groups, both orbits were found to be of the microseme type; no statistically significant difference was found (p > 0.05) (Table 3). The mean interorbital width was 2.63 ± 1.23 cm, and the biorbital width was 10.37 ± 5.51 cm. When interorbital/biorbital distances were evaluated comparatively between gender groups, both were found to be higher in males than in females, but the difference was insignificant (p > 0.05) (Table 4). Age-related changes in orbital width (Table 5), orbital height (Table 6), and OI (Table 7) were evaluated. In all age groups, these parameters were higher in males than in females, but the difference was statistically significant (p < 0.05) in one age group (10-19 years) only. Furthermore, when laterality was considered, all these parameters were higher on the right side than on left in all age groups irrespective of sex; however, the differences were significant (p < 0.05) only in one age group (10-19 years).

**Table 2:** Dispersion of orbital width and height according to left/right sides and genders.

Gender	Right height (cm)	Left height (cm)	p-value	Right width (cm)	Left width (cm)	p-value
Male (107)	3.46±1.90	3.25±1.60	0.75	4.33±2.05	4.30±2.04	0.91
Female (93)	3.38±1.60	3.20±1.50	0.82	4.30±2.02	4.26±2.01	0.89
Combined (200)	3.40±1.80	3.23±1.40	0.46	4.31±2.04	4.27±2.02	0.89

**Table 3:** Dispersion of orbital indices according to left/right sides and genders.

Gender	Right orbital index	Left orbital index	p-value	Combined orbital index
Male (107)	81.78±4.90	81.00±5.60	0.27	81.42±5.10
Female (93)	81.23±6.10	80.53±4.90	0.35	80.78±5.50
p-value	0.48	0.52	-	0.39
Combined (200)	81.55 ± 5.30	80.75±4.80	0.046	81.22±5.20

**Table 4:** Distribution of interorbital and biorbital widths according to genders in different age groups (r: Pearson’s correlation coefficient).

Age group (years)	Male (n = 107)			Female (n = 93)			Combined (n = 200)		
	Interorbital width (cm)	Biorbital width (cm)	r	Interorbital width (cm)	Biorbital width (cm)	r	Interorbital width (cm)	Biorbital width (cm)	r
10-19	1.87±0.03	9.82±0.01	0.23	1.81±0.01	9.71±0.01	0.30	1.84±0.03	9.77±0.07	0.27
20-29	2.11±1.12	10.13±0.17	0.04	2.07±1.27	10.11±0.33	0.07	2.10±0.77	10.12±0.43	0.03
30-39	2.27±1.17	10.31±0.22	0.02	2.23±1.47	10.25±0.39	0.04	2.26±0.88	10.27±0.27	0.01
40-49	2.35±1.13	10.35±0.43	0.07	2.33±0.87	10.31±0.32	0.07	2.34±0.43	10.32±0.37	0.07
50-59	2.30±0.67	10.28±0.33	0.19	2.27±0.53	10.25±0.36	0.14	2.29±0.51	10.27±0.17	0.17

**Table 5:** Dispersion of orbital width among different age groups.

Age group (years)	Male (n = 107)			Female (n = 93)			Combined (n = 200)		
	Right	Left	p-value	Right	Left	p-value	Right	Left	p-value
10-19	3.85±0.01	3.82±0.01	<0.01	3.80±0.01	3.71±0.01	<0.01	3.82±0.02	3.77±0.03	<0.01
20-29	4.28±1.22	4.19±0.20	0.40	4.17±1.35	4.15±0.30	0.80	4.23±0.80	4.17±0.40	0.34
30-39	4.37±1.30	4.35±0.28	0.87	4.35±1.50	4.23±0.40	0.40	4.36±0.90	4.29±0.20	0.28
40-49	4.35±1.10	4.32±0.50	0.79	4.35±0.90	4.31±0.30	0.68	4.34±0.50	4.32±0.30	0.62
50-59	4.31±0.70	4.31±0.30	0.90	4.27±0.50	4.25±0.40	0.74	4.30±0.40	4.28±0.10	0.49

**Table 6:** Dispersion of orbital height among different age groups.

Age group (years)	Male (n = 107)			Female (n = 93)			Combined (n = 200)		
	Right	Left	p-value	Right	Left	p-value	Right	Left	p-value
10-19	3.17±0.01	3.12±0.02	<0.01	3.15±0.01	2.97±0.02	<0.01	3.16±0.03	3.05±0.032	<0.01
20-29	3.37±1.10	3.35±0.10	0.85	3.27±1.35	3.15±0.30	0.40	3.32±0.60	3.25±0.30	0.14
30-39	3.53±1.20	3.47±0.23	0.61	3.33±1.50	3.27±0.38	0.70	3.43±0.70	3.37±0.30	0.26
40-49	3.51±1.20	3.42±0.30	0.45	3.41±1.20	3.31±0.40	0.44	3.46±0.80	3.37±0.40	0.15
50-59	3.50±0.60	3.43±0.20	0.25	3.43±0.30	3.36±0.50	0.24	3.47±0.50	3.40±0.40	0.12

**Table 7:** Dispersion of orbital index among different age groups.

Age group (years)	Male (n = 107)			Female (n = 93)			Combined (n = 200)		
	Right	Left	p-value	Right	Left	p-value	Right	Left	p-value
10-19	80.52± 0.06	80.17±0.030	<0.01	81.67±0.40	81.33±1.40	<0.01	81.47±0.02	80.89±0.05	<0.01
20-29	82.84± 2.00	83.16±1.30	0.16	78.73±3.20	79.95±6.12	0.08	80.77±2.18	81.56±5.17	0.16
30-39	81.50± 1.50	81.84±2.50	0.22	80.77±4.13	79.77±7.21	0.23	81.67±3.54	80.81±6.53	0.22
40-49	83.11± 1.20	83.39±2.30	0.26	80.68±2.37	79.16±8.11	0.07	81.91±5.61	81.28±8.37	0.70
50-59	81.89± 1.10	82.06±2.10	0.45	81.20±4.18	79.58±8.23	0.10	81.55±4.27	80.83±7.81	0.39

## Discussion

Precise knowledge about the morphology and morphometry of the eye orbit is a prerequisite in physical anthropology and reconstructive surgery of the orbito-maxillary region [1,8]. The purposes of the current analysis were to assess the changes in shape and dimensions of the orbit regarding age and gender and to assess orbital asymmetry.

Anthropological studies have already revealed the role of the eye orbit in determining the population affinity [1]. In the present study, we have investigated the variations in the shape of the bony orbit depending upon age, sex, and ethnicity.

The orbital region displayed clear variability in its growth rate. As evident from the measured parameters (orbital height/width and interorbital/biorbital distances), this region keeps on changing as age advances. However, according to Waitzman et al. [12], the interorbital region changes relatively little after birth, while the lateral wall of the orbit continues to grow throughout childhood, producing a wider orbit in adults. In the present study, we found rounded orbit in the pubertal age group (10-19 years), but later changes to elliptical and rectangular. Such changes in shape are not an abrupt phenomenon; it rather happens gradually. The main evolutionary tendency behind this is vertical compression and horizontal elongation of the orbit [1]. However, this tendency seems to be reversed from the current geological epoch, and the orbit tends to be relatively taller, narrower, and generally more rounded [1,13].

We have also reported variations in the contours of the orbit. The inferior contour of the orbit, formed by the maxilla and zygoma, showed maximum variability, followed by the medial margin, whereas the superior contour, formed by the frontal bone, showed the least variability. In agreement with our observations, Xing et al. [14] also reported greater variability on the bones of the maxilla and zygoma that form the inferior contour of the orbit, compared with the frontal bone forming the superior contour. It is hypothesized that the differential rate in the growth and resorption of the facial skeleton (frontal, maxilla, and zygoma) is responsible for such variations in the eye orbit contours. Cameron [15] noted that the orbit contours of people from Eurasia have more rounded corners than those of Africans, while Masters [1] showed that European specimens possessed orbital shapes similar to those of Africans. The anatomically modern Eurasian has commonly been characterized by a wide rectangular orbit [16].

The shape of the eye orbit has been used in some studies as a sex-distinguishing characteristic. Brown and Maeda [13] found that the female Australian Aborigines and Tohoku Japanese have relatively higher orbits than the males. Pretorius et al. [17] demonstrated the effective use of orbit shape as a sexually dimorphic trait in Bantu-speaking South Africans. In the present study, both males and females showed rounded orbits in the pubertal age group (10-19 years); however, as the age advances, we could establish sexual dimorphism. With advancing age, the orbits of males became square and rectangular, whereas female orbits became elliptical (elongated transversely).

Various authors have studied the regional variations in the shape of the orbital aperture. According to Cameron [15], the orbit of a contemporary adult human is generally quadrangular in shape but varies regionally in the curvature of the four corners. Most studies on orbit shape mainly involved facial skeletons from East Asia, Europe, and Africa. Studies showed clear discrepancies while describing orbit shape. Lahr [18] stated that the orbit shape of East Asians was too variable to be generalized. However, a taller, narrower, and more rounded orbit was found to be highly common for East Asian individuals, and this was supported by a series of studies [13], whereas Liu et al. [16] and Lu [19] have suggested an elliptical or square orbit aperture for Chinese populations [16,19]. Villiers [20] proposed a hypsiconch and rectangular orbit to be characteristic of Bantu-speaking South African populations. Masters [1], however, proposed that Africans were characterized by a much shorter orbit. The present study, performed on frontal radiographs of North Indian individuals, showed rounded orbits as the most common variant, closely followed by elliptical orbits.

Our radiographic study presented various aspects of orbital morphometry from a sample of 200 frontal digital radiographs of North Indian individuals. According to the study results, there was no significant difference between the right and left orbits in all the parameters studied (orbital height, width, and OI). This was in agreement with the findings of various authors such as Bentley et al. [21], Haas et al. [22], and Sforza et al. [23]. These results allowed us to combine both sexes during the analysis. Furthermore, orbital parameters did not show significant differences between males and females on both sides of the face. This was in agreement with the findings of Bentley et al. [21].

Thus, this study established the fact that the mean values of all parameters was higher in males than in females, irrespective of side, and was higher on the right side than on the left, irrespective of sex, but the difference was nonsignificant in both scenarios.

The OI was computed from the parameters obtained during the investigation and was found to be higher on the right side than on the left. The difference observed between the right and left sides could be attributed to the differential growth of the two sides of the brain. In this case, the right side has shown dominance, a factor that must be considered in the surgical correction of the bony orbit to ensure an efficient structural disposition of the visual apparatus [24].

The present study showed that the OI of the North Indian population was 81.65, which falls in the microseme category. This was in agreement with the earlier study described by Cassidy [7] for the black race and Kaur et al. [25] for the North Indian population. According to Cassidy [7], the microseme category might be an outcome of environmental trends and an impact of time on the study population. The transformation of the facial skeleton into the adult form is multifactorial. Although the basic structure of the orbits is genetically determined while in utero, postnatal modification occurs through functional matrices in response to environmental and epigenetic influences such as climate, activity patterns, and masticatory functions [26].

In our study population, the mean OI was higher in males (81.55 ± 5.30) than in females (80.75 ± 4.80) without any significant gender difference. On the contrary, Weaver et al. [25] showed that the OI was significantly higher in males than in females. On a few occasions, the OI was found to be higher in females than in males [11].

This study also demonstrated the mean values of the orbital parameters for various age groups in both sexes. Our findings showed an increase in the mean value of orbital parameters and eventually in OI with an increase in age up to age group 30-39 years when a peak was attained. Then there was a smooth decline in the mean values with a further increase in age.

Jeon et al. [27] studied the age-related changes of the orbital rim in Koreans and reported that the orbital aperture area did not show significant change with increasing age in either males or females. They further commented that changes that occur with advancing age were irregular with a combination of decreased and increased components.

Our findings on variations of the OI with age support the work of Igbigbi and Ebite [28], who previously studied the OI among adult Malawians. According to Parfitt [29], such a pattern either indicates genetically determined continuous variables like height or physique or might be due to continuous bone resorption and remodelling occurring at the cortical surface of the bone at regular intervals of two to five years while bone turnover for the whole skeleton occurred about 10% per year. In males, the smallest OI was 80.17 ± 0.03 and occurred in the age group of 10-19 years. However, in females, the smallest OI was 78.73 ± 3.20 and occurred in the age group of 20-29 years. When the sexes were combined, the smallest OI came out to be 80.77 ± 2.18 in the age group of 20-29 years. Ezeuko et al. [10] studied OIs of the Nigerian population aged between 0 and 79 years and found the smallest OI in the age group of 0-9 years. In total contrast, the smallest parameters were found in later age groups in the Malawian population [28]. This could be attributed to more chronic bone resorption and remodelling as a result of aging among adult Malawians. This is, however, subject to further studies.

In the present study, the OI of males attained a peak of 83.39 ± 2.30 at the age group of 40-49 years. However, in females, the OI attained a peak of 81.67 ± 0.40 at the very early age group of 10-19 years. Contrary to our study, the OI attained a peak at the later age group (40-49 years) in the Nigerian population [11]. When sexes were combined, the OI attained a peak of 81.91 ± 5.61 at the age group of 40-49 years. In Malawians, the OI attained a peak in the age group of 48-57 years in both sexes [28]. In the Nigerian (Igbo) population [10], the OI attained a peak slightly earlier (30-39 years). This earlier peak among the Igbos could be an indication of early metamorphic changes in the bones of the orbit. However, this hypothesis is subject to further study.

We have attempted to correlate the morphometric parameters of the orbits with the age of the subjects. In most of the age groups, no significant relation was found except in one age group (10-19 years).

Ezeuko and Om'Iniabohs [10] also studied the age changes of the OI in different age groups. They found no significant differences in OI between males and females of the age groups of 20-29, 40-49, and 50-59, while the OI of males was significantly higher than that of females in the age groups 0-9, 10-19, 30-39, 60-69, and 70-79 years.

When left or right sides were taken into consideration, no significant relation was found in most of the age groups except in one age group (10-19 years) on both sides. In contrast to our findings, Ezeuko et al. [10] found a nonsignificant difference between male and female right OI in age groups 0-9, 20-29, 30-39, 40-49, and 50-59 years, while in males, the left OI was significantly higher than the left OI in females in the age groups of 60-69 and 70-79 years on both sides.

The interorbital/biorbital distances were larger in males than in females, but the difference was nonsignificant, which was in agreement with the findings of Kaplanoglu et al. [11].

Schmittbuhl and Le Minor [30] studied 44 skulls (32 males, 12 females) to define and quantify the relative positions of the orbital and nasal apertures in the human face. They have defined significant proportions of the human face by comparing the interorbital and nasal width with biorbital width. On average, the interorbital width corresponded to a fifth of the biorbital width and thus to half of the mean orbital width. In our study population, interorbital distance corresponded to less than a fifth of the biorbital width in males, more than a fifth of the biorbital width in females, and a fourth of the biorbital width when combined. Correlation analysis showed no significant relationship between interorbital/biorbital distances in all age groups.

## Conclusions

Orbital dimensions showed no association with age and gender except in one age group (10-19 years). A pubertal growth spurt in females might be causing this phenomenon. The morphometric data may be useful in forensic anthropology and better planning for reconstructive surgery in the orbito-maxillary region. We conclude that the differences in orbital morphometry derive more from ethnic diversities than from age and gender. Such studies seem to have great potential relevance in anthropology and clinical biometry.
